# Supplementary material for: Cryo-EM structure of the conjugation H-pilus reveals the cyclic nature of the TrhA pilin
Source: bioRxiv. 2024 Dec 31:2024.12.30.630807. Preprint. [Version 1] doi: 10.1101/2024.12.30.630807 (PMC11722321; doi:10.1101/2024.12.30.630807)
Supplement: 1 [file NIHPP2024.12.30.630807V1-supplement-1.pdf]

Supplementary Table 1. Strains, plasmids and primers used in the generation of the MG1655 Δ*trp* and Δ*fimA* mutants.

**Strain**	**Description**	**Resistance**
MG1655	*Escherichia coli* K12 strain (template for directed mutagenesis of *trp*- mutant)	-
CC118 λpir	Triparental mating donor strain	-
E.coli pRK2013	Triparental mating helper strain	Kanamycin

**Plasmid**	**Description**	**Resistance**
R27	IncH1 conjugative plasmid	Tetracycline
drR27	Derepressed R27 plasmid in which resistance was swapped from tetracycline to chloramphenicol	Chloramphenicol
pSEVA612S	Tri-parental mating mutagenesis vector	Gentamycin
pACBSR-Sm	Lambda red fragment expressing plasmid.	Streptomycin

**Primer**	**Sequence (5'→3')**	**Description**
pSEVA612s vector_fwd	tttccacggtTAGGGATAACAGGGTAATTAC	Amplifies linear pSEVA612S
pSEVA612s vector_rev	gcaacgcgcaTAGGGATAACAGGGTAATC	
down trp HR_fwd	gttatccctaTGCGCGTTGCGGATCATTTTTAAAATTAC	Amplifies downstream Homology region flanking *trp* operon
down trp HR_rev	gagaataacaTCCCACAGCCGCCAGTTC	
up trp HR_fwd	ggctgtgggaTGTTATTCTCTAATTTTGTTCAAAAAAAAG	Amplifies upstream homology region flanking *trp* operon
up trp HR_rev	gttatccctaACCGTGGAAATTTCCACG	
B.ISceI	TAGGGATAACAGGGTAAT	Amplifies linear pSEVA612S
fim HR1_fwd	GGATTACCCTGTTATCCCTAcatggcgtaagctgacgaatc	Amplifies upstream Homology region flanking *fim* operon
fim HR1_rev	agcaatgtcctgtgatttctcgtttttcccttataattacagacg	
fim HR2_fwd	gtaattataagggaaaaacgagaaatcacaggacattgctaatgctg	Amplifies downstream homology region flanking *fim* operon

Supplementary Table 2. Data collection and processing parameters, and refinement statistics.

	H-pilus
**PDB entry**	9HVC
**EMDB entry**	EMD-52431
**Data collection and processing**	
Magnification	130,000
Microscope	Titan Krios G2
Voltage (kV)	300
Detector	Falcon 4i
Electric exposure (e^-/Å)	50
Defocus range (μm)	-0.8 to -1.8
Pixel size (Å)	0.921
Data Processing Program	cryoSPARC (v.4.6.1)
Movies	869
Initial / Final particle images (no.)	492,091 / 123,942
Symmetry imposed	C5
Helical rise (Å)	12.14
Helical twist (°)	28.93
Map resolution (Å)	2.2
FSC threshold	0.143
**Refinement**	
Refinement Program	PHENIX (v.1.20.1)
Map:model resolution (Å)	2.2
FSC threshold	0.5
Model composition	
Non-hydrogen atoms	578
Protein residues	69
Water	31
R.m.s. deviations	
Bond length (Å)	0.003
Bond angles (°)	0.388
Validation	
MolProbity score	1.22
Clashscore	4.45
Ramachandran plot	
Favored / Allowed (%)	100 / 0
Disallowed (%)	0.00
Mask CC	0.88

Supplementary Figure 1

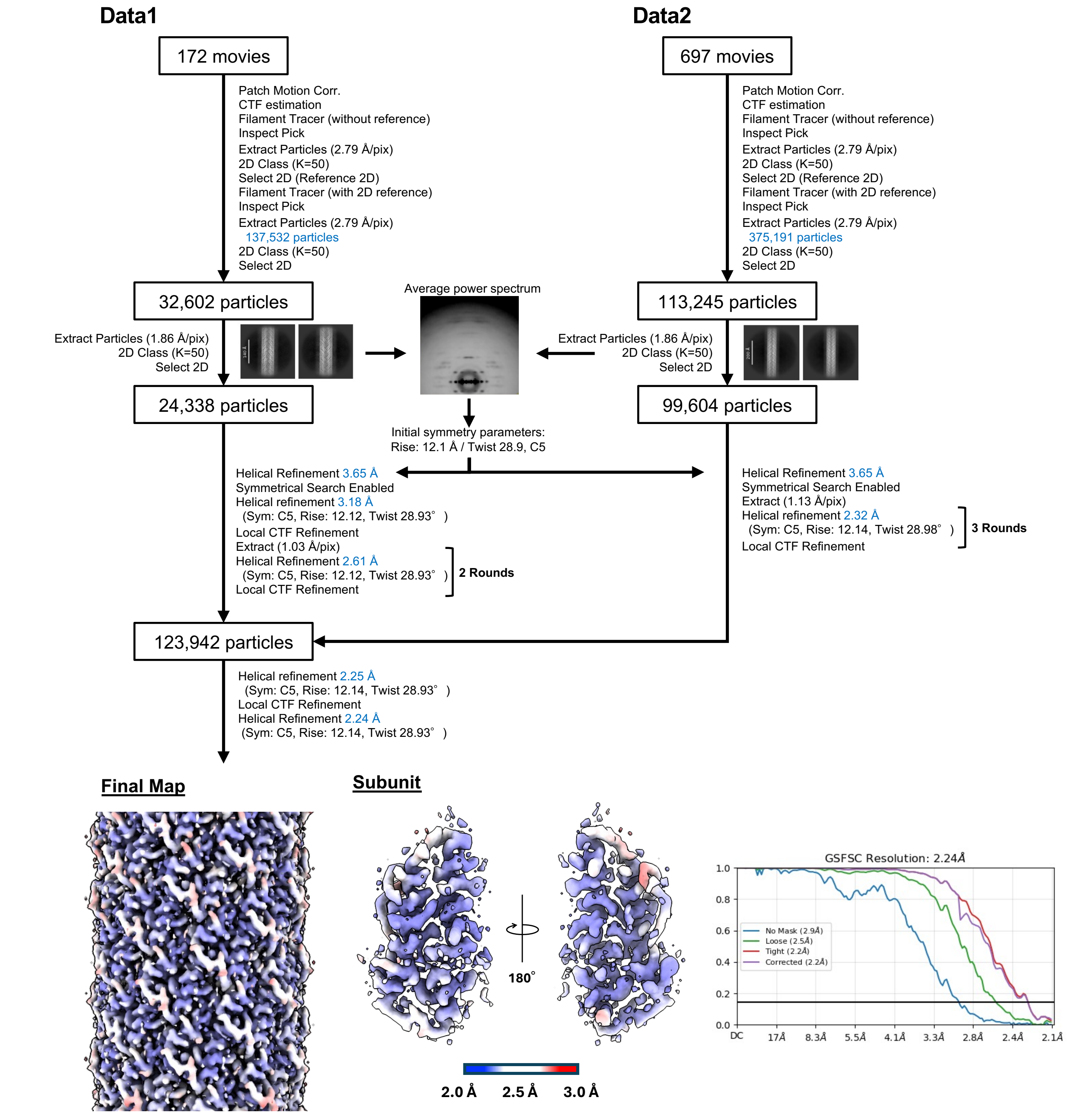

Supplementary Figure 1. Cryo-EM data processing workflow
Data processing workflow for H-pilus. All the processing was performed in cryoSPARC (v.4.6). The Final map was colored according to the local resolution. Gold-standard FSC curve of the final map is shown. The resolution was determined at FSC=0.143.

Supplementary Figure 2

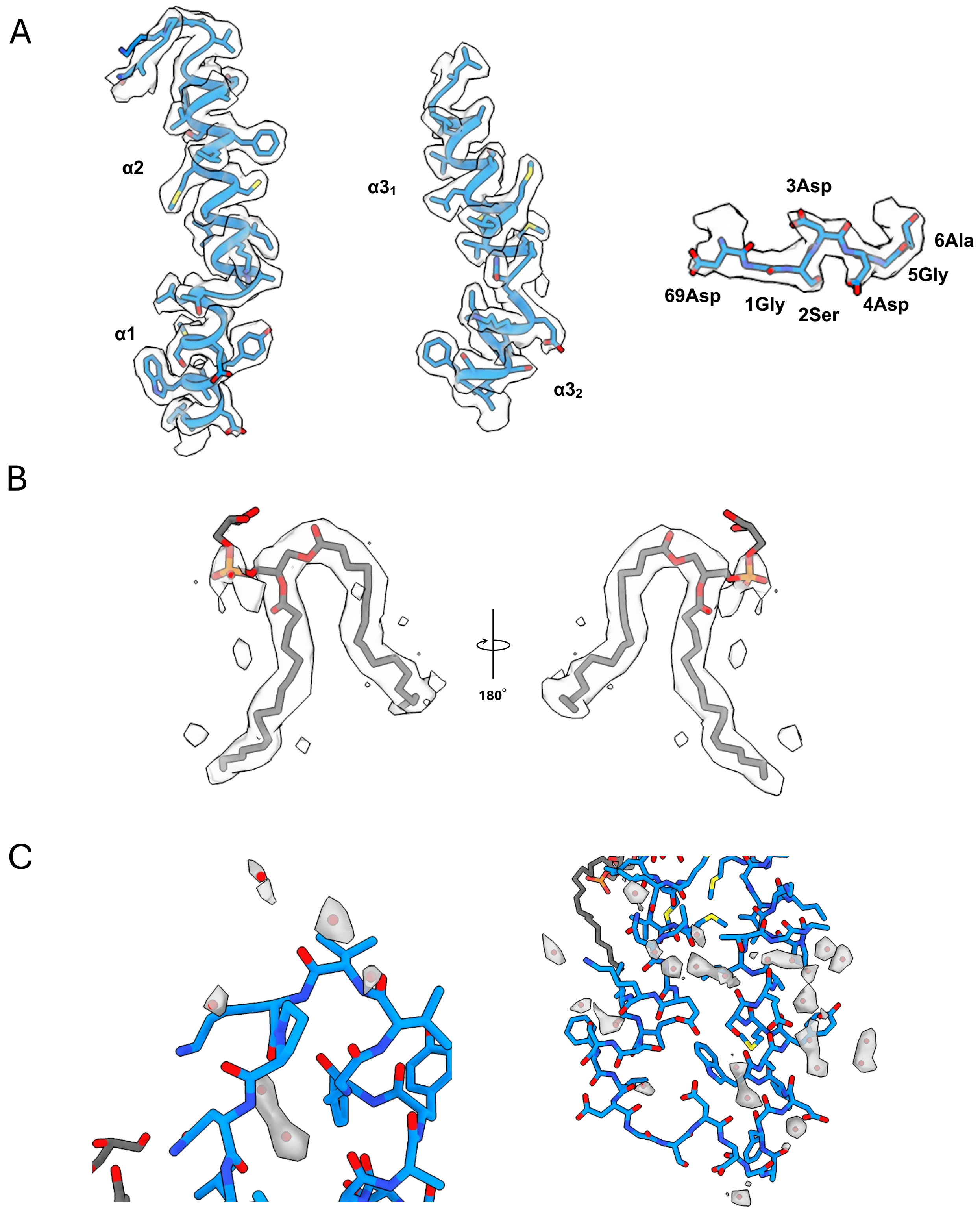

Supplementary Figure 2. Cryo-EM density map and models.
The map and models showing: (A) helices α1,2,3 and cyclization region; (B) lipid; and (C) water molecules.

Supplementary Figure 3

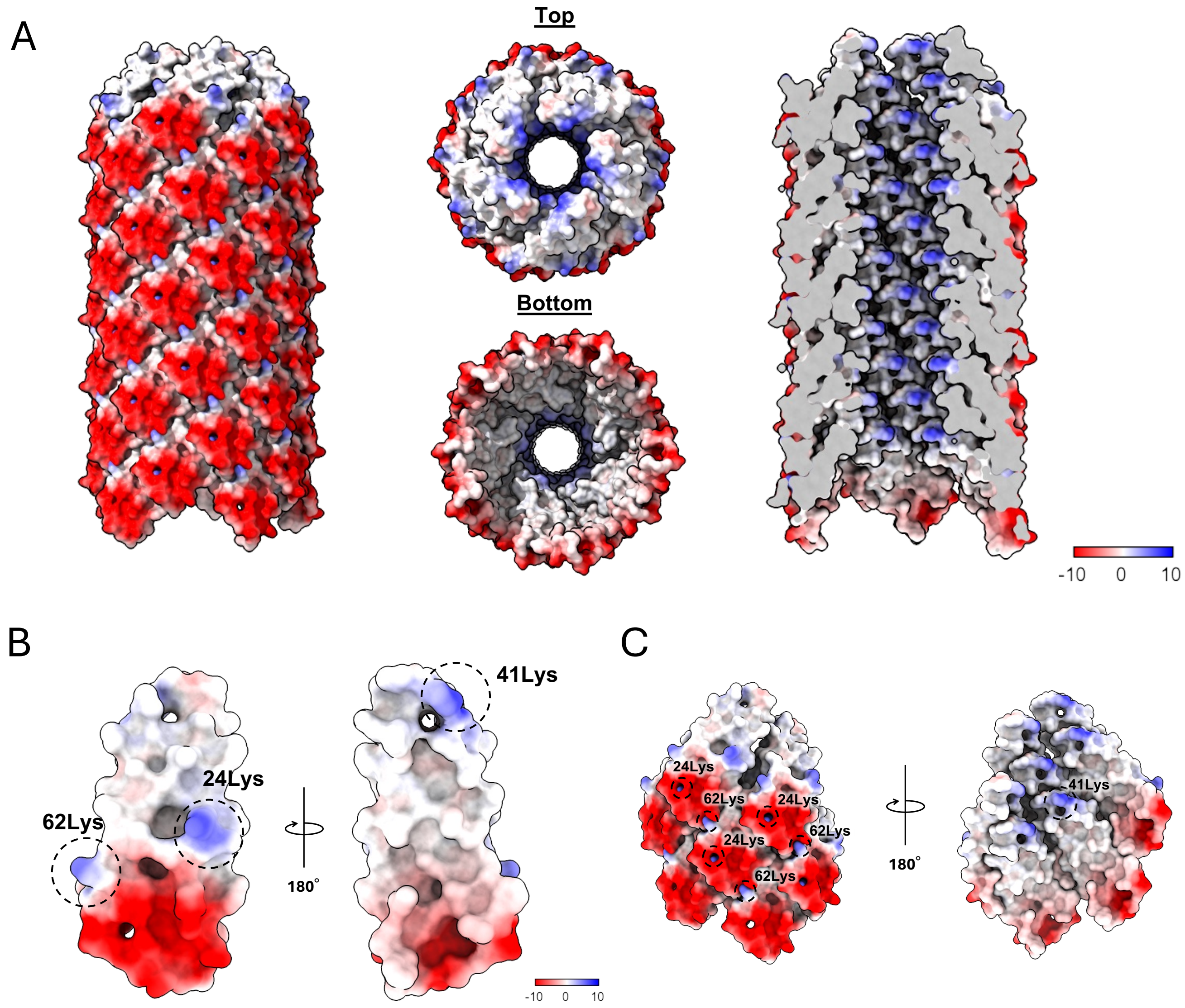

Supplementary Figure 3. The surface electrostatic potential of H-pilus. All potentials have been calculated without the bound lipid.

(A) The potential is shown in the side view (left), top and bottom views (middle), and cutaway view (right). The electrostatic potential is colored from red (negative) to white (neutral) to blue (positive).

(B) A detailed surface electrostatic surface of the TrhA pilin is shown in two orientations. Key lysine residues (Lys24, Lys41, and Lys62) are highlighted with dashed circles.

(C) Residues in neighboring subunits are labelled, and analysis of electrostatic interactions between subunit and subunit. The view is shown in two orientations, highlighting key lysine residues.

Supplementary Figure 4

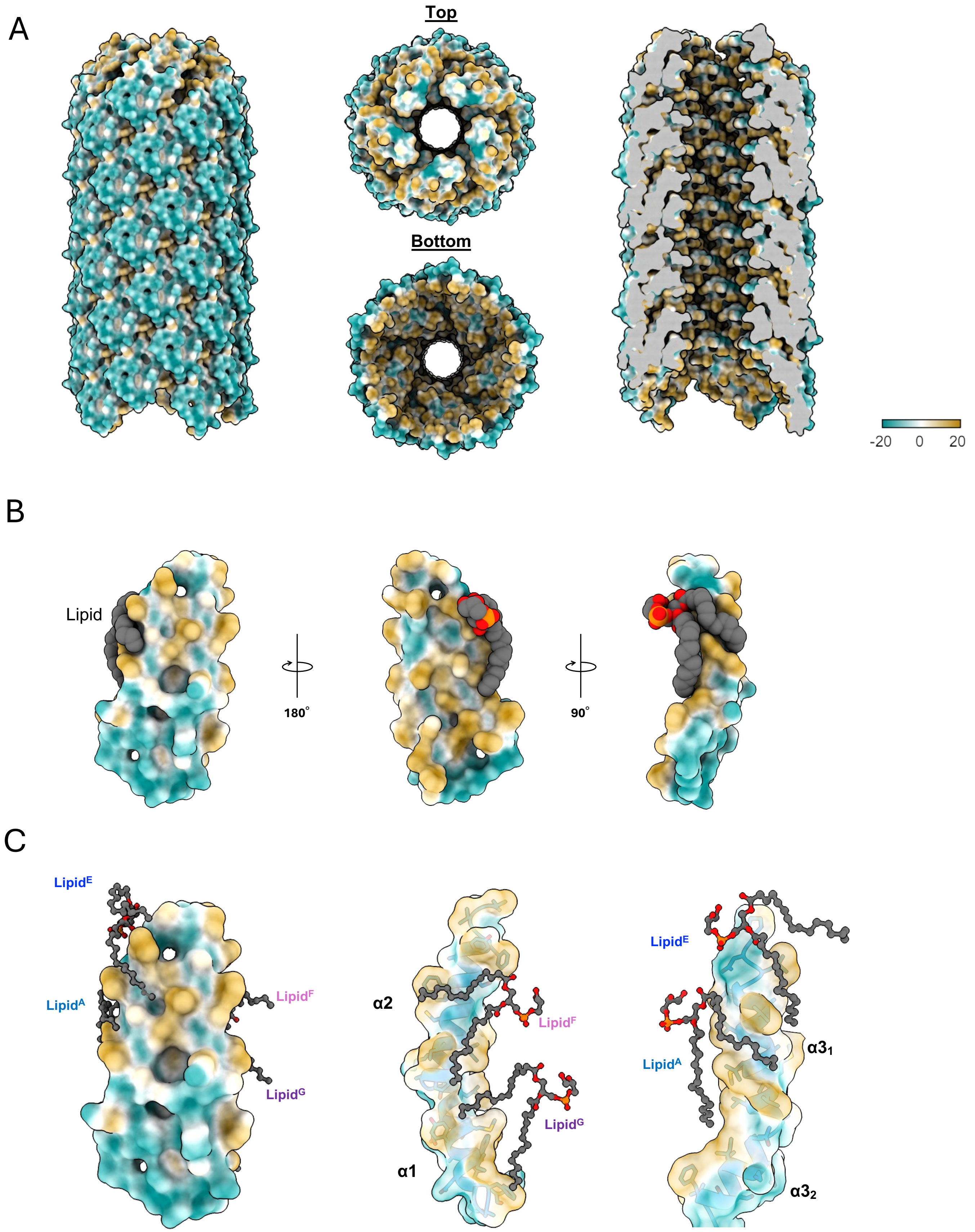

Supplementary Figure 4. The surface hydrophobicity analysis of H-pilus.

(A) The surface hydrophobicity is shown in side view (left), top and bottom views (middle), and cutaway view (right). The hydrophobic potential is colored from gold (hydrophobic) to white (neutral) to cyan (hydrophilic).

(B) Surface hydrophobicity of the TrhA pilin and its association with the PG lipid is shown in three orientations.

(C) Surface hydrophobicity of lipid-binding pockets in α1, α2, and α3.
